# Remarks on “Snuff Chewing,” and a Mode in Which It May Affect the Pulmonary Organs

**Published:** 1850-07

**Authors:** R. A. Kinloch

**Affiliations:** Charleston


### REMARKS ON "SNUFF CHEWING," AND A MODE IN WHICH IT MAY AFFECT THE PULMONARY ORGANS.

BY R. A. KINLOCH, M. D., CHARLESTON

Reader, judge not from the caption of this article, that I am about to prosecute the war so long, vigorously, and continuously waged against the fascinating weed you so much love: no, for could such a war avail, its object of assault would long since have ceased to exist but in history. Never has a poor plant, deriving nourishment from the earth, been more condemned or more eulogised. To its use has been traced the origin of "all the ills which flesh is heir to." Physicians have never ceased to preach of its abuse. Kings have felt it obligatory upon them to banish it from their realms—nay, the church has raised her strong arm; and the anathemas of the "Holy See" been hurled against those who would attempt a "*pinch of snuff*" within the precincts of the temple of the "Most High." The universality of its employment, however, at present, throughout the world, attests the little good which has resulted from the opposition. In every clime, how many are still ready to exclaim with Burton:—"Tobacco, rare, superexcellent, divine tobacco, which goes far beyond all their panaceas, portable gold, and philosopher's stones, a sovereign remedy for all diseases!"

'Tis not then against "*tobacco*" I fight; though I disapprove of its habitual use in any shape; but *tobacco in the form of snuff*, applied habitually to the buccal mucous surface, and not the nasal, as is more common. I lay aside, also, its deleterious effects, as an acrid narcotic vegetable, upon *this* mucous surface, and its more remote action upon the digestive organs, and the nervous system; for these have already been fully expatiated upon, and the result of much investigation placed long before the profession.

My object, in the present paper, is simply to call the attention of the profession to the possibility of the article—tobacco—in its minutely divided state, being received directly into the lungs, with the inspired air; and, there collecting, prove an efficient predisposing and exciting cause of disease and disorganization of the pulmonary organs; and, that this is more apt to ensue from the disgusting habit of introducing the snuff into the mouth for chewing, than from snuffing, in the ordinary acceptation of the term.

This truly revolting habit is, happily, much the less popular of the modes in which tobacco is abused, and what is remarkable, we find it almost exclusively confined to the fairer sex. I am aware of no instance of the habit in the male. "Could any one," says Dr. Ticknor, in his 'Philosophy of living,' "entirely unacquainted with the unaccountable habits and propensities of man, after knowing the properties of tobacco, be told that America's fair daughters use it too, would he not be perfectly incredulous? And were he told, further, that ladies of the greatest respectability, the most genteel and accomplished in one of our largest cities, carry their little jars or boxes of snuff into the social circle, and with a delicate ivory spoon feed their *sweet* mouths with this most delicious and agreeable poison, would he not be petrified with astonishment?" Yet, 'tis sadly true! Reason is almost at a loss to account for so vicious a propensity. In one instance which passed under my notice, a beautiful woman, from Maryland, who acquired the habit whilst on a visit to Florida, the practice was induced from using the artical as a dentifrice. Apart from this, I can offer no plausible account of the fancy of the fair ones for so strange a habit, any more than I can explain "how, in obedience to fashion, they defy physical laws, breathe freely in armour, and feel warm in gossamer."

Now it is a matter of every day experience, that the attempt to take into the mouth any substance in a state of minute division, is frequently productive of a spasmodic action of the glottis—we have a short convulsive cough—the foreign body has been conveyed within the glottis, during an inspiration, which was involuntary; and, now, as an irritant, is productive of these reflex actions which are conservative to the part—their result being, generally, the expulsion of the foreign particles. But though such is, in general, the result, from the nice sensibility of the mucous surface, it is by no means invariably so. We see it daily verified, that a mucous surface *may tolerate for a time*, the presence of a foreign body; and that the *pulmonary mucous surface* is amenable to the *general law*, the records of medicine afford ample testimony. Dr. J. C. Gregory, of Edinburgh, was the first to enlighten the profession upon this interesting point. He found, upon examination, the lungs of a patient, who died under his care, filled with a black carbonaceous matter; along with this there were marks of inflammation and disorganization of the pulmonary tissue; there were cavities containing a considerable quantity of black liquid. This patient had, for a number of years, been a worker in the coal mines; which fact, together with the chemical analysis of the black substance found in the lungs, pointed to this conclusion: *The black matter was of extraneous origin; and nothing more than the coal dust taken in with the inspired air!* The report of this case was, at once, the stimulus to investigation which proved, beyond doubt, that this was the discovery of a new truth! The same condition of things was found, after death, in the bodies of many others engaged in similar occupations; and, it was furthermore, observed that many of these individuals expectorated a "*black sputa*," during life. It has been, also, since discovered that the lungs of old persons, long resident in large cities, often present traces of a dark deposite, evidently derived from the habitual inhalation of a sooty atmosphere.

May not particles of tobacco then, if inhaled, collect to some extent, in a similar manner? That they gain access to the pulmonary mucous surface, as readily as particles of other kind cannot, of course be denied; but it will be at once objected, that, from the acrid irritating properties of this article, it must be soon expelled: or, if remaining, be productive of constant and harrassing pulmonary symptoms—that tolerance for a time even, cannot be established. This is certainly a reasonable, natural conclusion; and the one which I was diposed to adopt, until the "*facts*"—those "stubborn things"—of the case below, seemed more convincing than *a priori* reasoning.

On *April 28th*, 1949, I was requested to see Jane, a colored girl, aged 28, of tolerable good general health up to this period. Found her with "hemopthisis." She had raised several ounces of blood and mucus this morning, before I was sent for. She had now a cough, and continued to expectorate small quantities of blood. Pulse somewhat frequent, but not full. Percussion gave some dullness over the posterior and middle portions of the right lung; auscultation, "large crepitation" in the same region. Respiration good throughout left lung; sounds of heart, normal. Directed to be purged with salines, and afterwards to take nauseants, with an anodyne; stimulating applications to extremities. Second visit:—condition much the same; and now, upon examining closely the expectoration, was surprised to see *numerous dark particles diffused through it.* In some spots, several of these had coalesced and formed large masses. While looking more attentively, to decide what these might be, the mother of the patient asked me if this was not snuff she had been spitting up?

Upon my answering that it looked like it, and inquiring if the patient used the article, she informed me that "*she had been in the habit of 'chewing snuff' for about six years!*" That it was a "*fashion*" with the colored girls of her acquaintance; and that she could not persuade her daughter to abandon it, though, for some time she had experienced bad feelings, which she attributed to the vice. I thought the matter now explained. The snuff had come from the mouth! But upon inquiry, I was assured by the patient that "*she had not touched the article for four days!*" She had been feeling so unwell as to make it seem necessary to desist. Her mother, whom I have always found to be an honest, good woman, and who was much opposed to this vicious habit of her daughter, thought she could not possibly have used the snuff within that time, as she had been under her eye—having moved home to her room to be nursed. I examined the mouth, fauces and nostrils of the patient, but could detect nothing contradictory to her statement. No snuff was to be found about the patient, the bed or the room. I directed kreasote to be added to the nauseant and expectorant mixture; had the vessel containing the sputa emtied; and left, after warning them of the "*terrible results*" which might ensue if she continued to use the snuff in her present condition. (This was done to alarm them, if, perchance they had deceived me.)

*April 29th.*—The hemorrhage had gradually lessened; but *snuff was still diffused through what was brought up, and continued so the whole of this day and the next.*

*May 1st.*—Patient expressed herself much better; and, this day, the sputa was but slightly tinged with blood; could observe no particles of snuff. Directed to continue mixture of kreasote, &c.

*May 4th.*—She felt so well that she ventured to leave her room. The consequence of this was, I found her on the morning of the fifth with symptoms of "pneumonia," which disease, in a day or two, in spite of remedies used, was fully developed. There was now the usual rust-colored sputa; auscultation gave the fine peculiar crepitation, where before I had heard the large; and the accompanying fever was of a low grade. Her condition grew gradually worse, and for some time, promised an unhappy end. Calomel, opium and ipecac, together with the stimulating expectorants, flying blisters to the chest, &c., were now resorted to. These means succeeded in turning a favorable balance. A gentle ptyalism was established, and soon followed by a speedy convalescence. She, in a short time, regained her usual health, except that there was an occasional cough. I lost sight of her from that time until the present month—almost a year—when I was called to prescribe for her child. She is now looking very well, but complains of being "sick, off and on, with a cough," and has "several times raised blood." She has continued to chew snuff since her recovery from the severe sickness, and expresses inability to leave it off, though believing it has injured her. I proposed a compromise, i. e., to substitute tobacco for snuff. But, "no;" *this is not feminine!* I ascertained from her, now, that the attempt to introduce a spoonful of snuff into the mouth has, from the first, been frequently followed by a convulsive cough; and in relation to her companions addicted to the vice, that the *friend* from whom she learnt the habit, is troubled with a constant cough, and from having been a strong, hearty girl, has become emaciated and weakly.

I have, in the small sphere of my reading, seen the report of no case similar to the above; and, indeed, in but one place do I find the possibility of such an occurrence hinted at. Dunglinson, in his "Human Health," speaking of the common remark, that those engaged in the preparation of tobacco are subject to numerous infirmities, says: "It is probable that the mischief, if any result, is produced more by the small particles diffused in the air, in one of these processes—snuff-making—than from any narcotic influence of the plant; *and hence, we could comprehend that snuff-makers might, like millers, glass-cutters, &c., be exposed to lung affections, owing to the fine particles entering the lungs during inspiration, and exciting obstructions in these organs.*" (The italics are our own.) He finds, however, in the report to the Minister of Public

Works of France, nothing confirmatory of his view; for it is asserted, that in the manufactories of snuff in France, in which five thousand workmen are employed, they seem to become "*habituated*" to the atmosphere. Nay, the reporter even makes the working in tobacco *prophylactic of phthisis*, as well as the prevalent epidemics! Ah! Mon. Simeon, (the authority quoted,) we verily fear you are of the Burton School! But that the workmen became "*habituated*" to the atmosphere, implies, necessarily, that it was more or less irrespirable at first—the particles did enter and vex the respiratory organs! Now, Dr. Gregory's patient had become, we presume, "*habituated*" to the atmosphere of the coal mine; yet, the scalpel, after death, told a tale that was never dreamed of.

I would not, however, be understood as indulging, for a moment, the idea that the lungs may tolerate tobacco as readily as any other substance. The only inference I draw is, that sufficient accumulation may take place to prove an existing cause of disease. But it may be advanced, that as we, *in general*, respire through the nostrils, the foreign particles, of whatever kind, derived from the atmosphere, must, should they gain access to the lungs, enter chiefly through this channel; and *ergo*, "snuffing," which is much more frequently practiced than the "chewing of snuff," would be more likely to present us with pulmonary disease. To this, I reply: First—That our respiring, *in general*, through the nostrils, by no means proves that the larger proportion of particles gain access in this way; for, as we also respire by the mouth, one inspiration through this way may be productive of as much harm as many through the other; and I am inclined to think that it is through this more direct and extensive channel—the mouth—that one may suffer in a dusty atmosphere. At all events, it is certain, that when in such an atmosphere, we are soon cognizant of the foreign matter having entered the buccal cavity. Secondly—Though much of the dust in a coal mine may, and probably does enter through the nares, this is not so apt to be the case with the tobacco dust—if I may so call it—which is coarser, and more likely to come in contact with, and be detained by, the walls of so narrow and indirect a passage. Thirdly—I cannot believe that "snuffing" occasions the ingress of many particles into the lungs; for though a novice in the practice may find, upon taking a "pinch," that a portion has passed to the fauces, and perhaps into the glottis, from the disagreeable sensations experienced, yet, he soon learns to "snuff" with ease and pleasure—a voluntary and automatic movement of the muscles concerned prevent the progress of the snuff to the posterior nares. And fourthly—We have not been presented with a case similar to the above, as the result of the habit of "snuffing;" and so, until proved to the contrary, must conclude that, of the two habits, "snuff-chewing" is much the most likely to engender pulmonary disease.

